# Mitochondria‐Nuclear Crosstalk: Orchestrating mtDNA Maintenance

**DOI:** 10.1002/em.70013

**Published:** 2025-05-26

**Authors:** Ghazal Darfarin, Janice Pluth

**Affiliations:** ^1^ Department of Health Physics and Diagnostic Sciences University of Nevada Las Vegas Nevada USA

**Keywords:** anterograde and retrograde signaling, cellular homeostasis, mitochondrial biogenesis, mitochondrial dynamics, mtDNA maintenance, mitochondrial‐nuclear communication, redox signaling

## Abstract

The mitochondria (mt) and nucleus engage in a dynamic bidirectional communication to maintain cellular homeostasis, regulating energy production, stress response, and cell fate. Anterograde signaling directs mt function, while retrograde signaling conveys metabolic and stress‐related changes from mt to the nucleus. Central to this crosstalk is mitochondrial DNA (mtDNA), which encodes key oxidative phosphorylation components. MtDNA integrity is preserved through quality control mechanisms, including fusion and fission dynamics, mitophagy, and nuclear‐encoded DNA repair. Disruption in these pathways contributes to mt dysfunction, oxidative stress, and genetic instability—hallmarks of aging and diseases. Additionally, redox signaling and NAD+ homeostasis integrate mt and nuclear responses, modulating transcriptional programs that support mt biogenesis and stress adaptation. This review explores the molecular mechanisms coordinating mito‐nuclear interactions, emphasizing their role in maintaining mtDNA integrity and cellular equilibrium. Understanding these processes provides insights into how mt dysfunction drives aging and disease, paving the way for targeted therapeutic strategies.

## 1 Introduction

Communication between the nucleus and mt is bidirectional, ensuring cellular homeostasis through the continuous exchange of signaling molecules, proteins, and metabolites. This dynamic interaction is fundamental to mitochondrial DNA (mtDNA) maintenance, coordinating stress responses, metabolic adaptation, and cell survival (English et al. 2020; Monaghan and Whitmarsh 2015; Poyton and McEwen 1996). The nucleus exerts control over mt function through ‘anterograde signaling’, a process that regulates mtDNA replication, repair, and biogenesis in response to cellular demands (Whelan and Zuckerbraun 2013). This regulation is primarily mediated by nuclear‐encoded mt‐targeted proteins, such as transcription factor A mitochondrial (TFAM), peroxisome proliferator‐activated receptor gamma coactivator‐1 alpha (PCG‐1α), and nuclear respiratory factors 1 and 2 (NRF1/2), which collectively drive mtDNA replication and sustain mt integrity under both basal and stress conditions (Quirós et al. 2016; Savu and Moisoi 2022).

**FIGURE 1:**
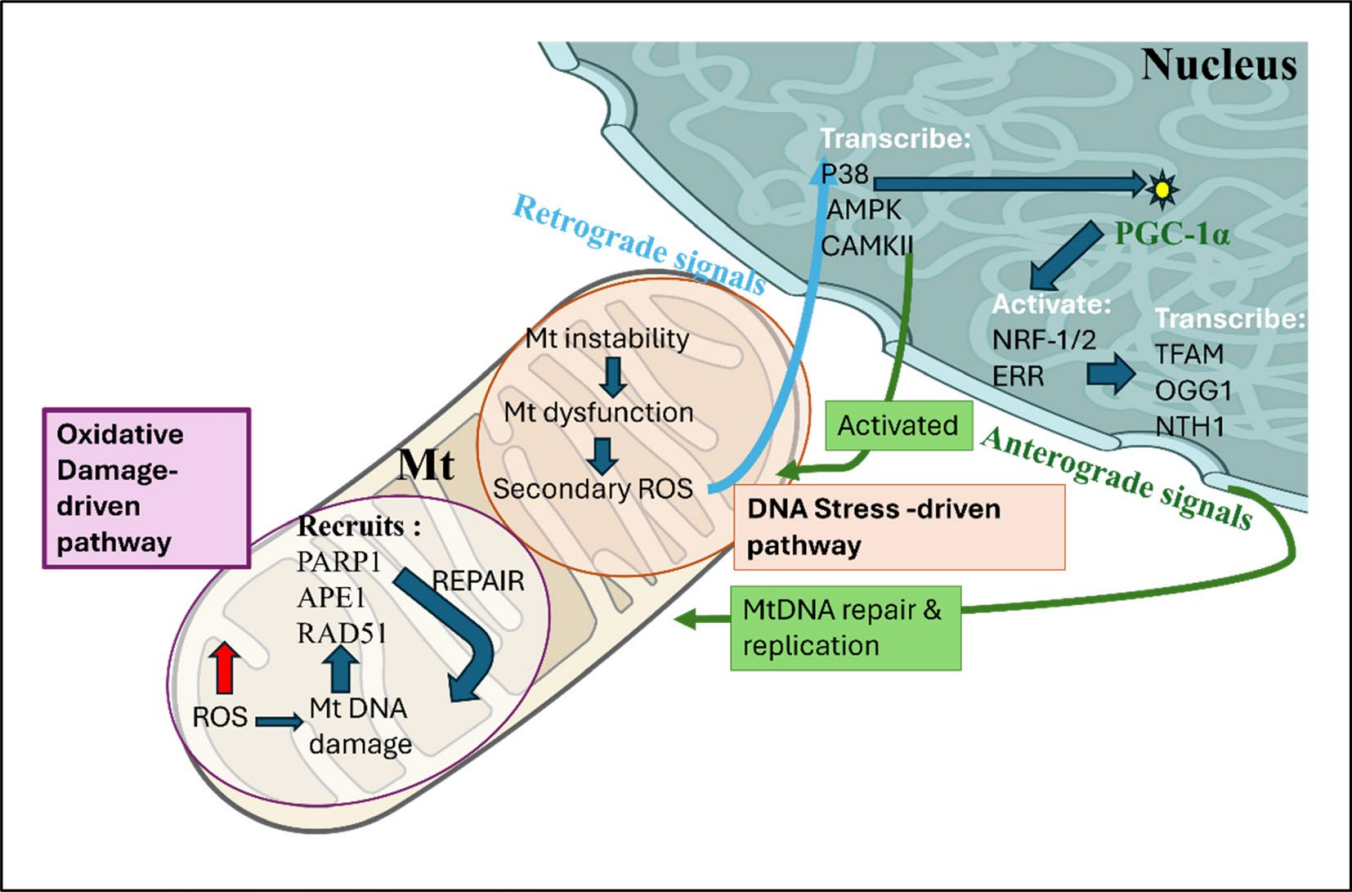
Bidirectional mito‐nuclear communication in mtDNA maintenance. Mt stress signals regulate mtDNA repair and biogenesis through retrograde and anterograde signaling. Two pathways coordinate this response. Oxidative damage‐driven pathway: mtDNA lesions caused by ROS activate PARP1, APE1, and RAD51, which are recruited to damaged mtDNA sites, promoting base excision repair (BER) and homologous recombination. The second pathway, the DNA stress pathway, mtDNA instability or replication stress triggers mt dysfunction, generating secondary ROS which initiate p38, AMPK, and CAMKII transcription through retrograde signaling and subsequent phosphorylation of PGC‐1α, enhancing its co‐activator function to promote transcription of NRF‐1/2 and ERR. This cascade drives the anterograde response of expression of TFAM, OGG1, and NTH1, which then mediates mt DNA repair and replication.

In turn, mt communicate its functional status back to the nucleus through retrograde signaling pathways, which transmit signals of mt dysfunction to reprogram nuclear gene expression. These pathways are often initiated by metabolic and oxidative stress cues, such as increased Ca^2+^, reactive oxygen species (ROS), or changes in NAD^+^/NADH ratios, triggering nuclear adaptations that restore mt function and promote cell survival (Jazwinski 2014; Walker and Moraes 2022). The coordinated integration of anterograde and retrograde signaling–referred to as mito‐nuclear communication–is essential for preserving mtDNA integrity, particularly under stress conditions such as radiation‐induced damage (Table 1, Figure 1) (Matilainen et al. 2017; Zhu, Li, et al. 2022).

Recent findings highlight how the exchange of metabolites between mt and the nucleus fine‐tunes cellular adaptation to environmental and metabolic stress. Mt‐generated ATP diffuses through the cytosol to fuel nuclear processes, while nuclear ATP can also be produced locally through glycolytic enzymes. In contrast, NADH does not diffuse directly between organelles but influences nuclear metabolism via the cytosolic NAD+/NADH pool, regulated by shuttle systems like the malate–aspartate shuttle. Acetylcarnitine shuttling further links mt acetyl‐CoA production to nuclear histone acetylation, integrating mt metabolism with gene regulation (Izzo et al. 2023). Additionally, certain mt proteins, such as superoxide dismutase 2 (SOD2), can relocate to the nucleus in response to stress, where they modify chromatin structure and transcriptional programs (Coelho et al. 2022).

NAD+ metabolism represents another critical axis of mito‐nuclear communication. Mt NAD+ depletion can profoundly impact nuclear transcription, particularly through sirtuin (SIRT)‐dependent epigenetic mechanisms that regulate stress adaptation and genome stability (Rigon et al. 2021). These emerging insights underscore the complexity of mt‐nuclear crosstalk, where the coordinated exchange of signals, proteins, and metabolites plays a pivotal role in cellular adaptation to environmental and genetic stressors.

This review delves into the molecular mechanisms that maintain mtDNA integrity within the framework of mito‐nuclear communication. We examine the pathways regulating mtDNA replication, repair, and quality control, emphasizing the cooperation between nuclear and mitochondrial networks in mtDNA maintenance. Additionally, we highlight emerging epigenetic modifications that facilitate cellular adaptation to physiological and stress conditions. Understanding these processes will provide critical insights into how mt dysfunction contributes to aging and disease, offering new avenues for therapeutic intervention.

## 2 Regulation and Dynamics of mt Maintenance

**FIGURE 2:**
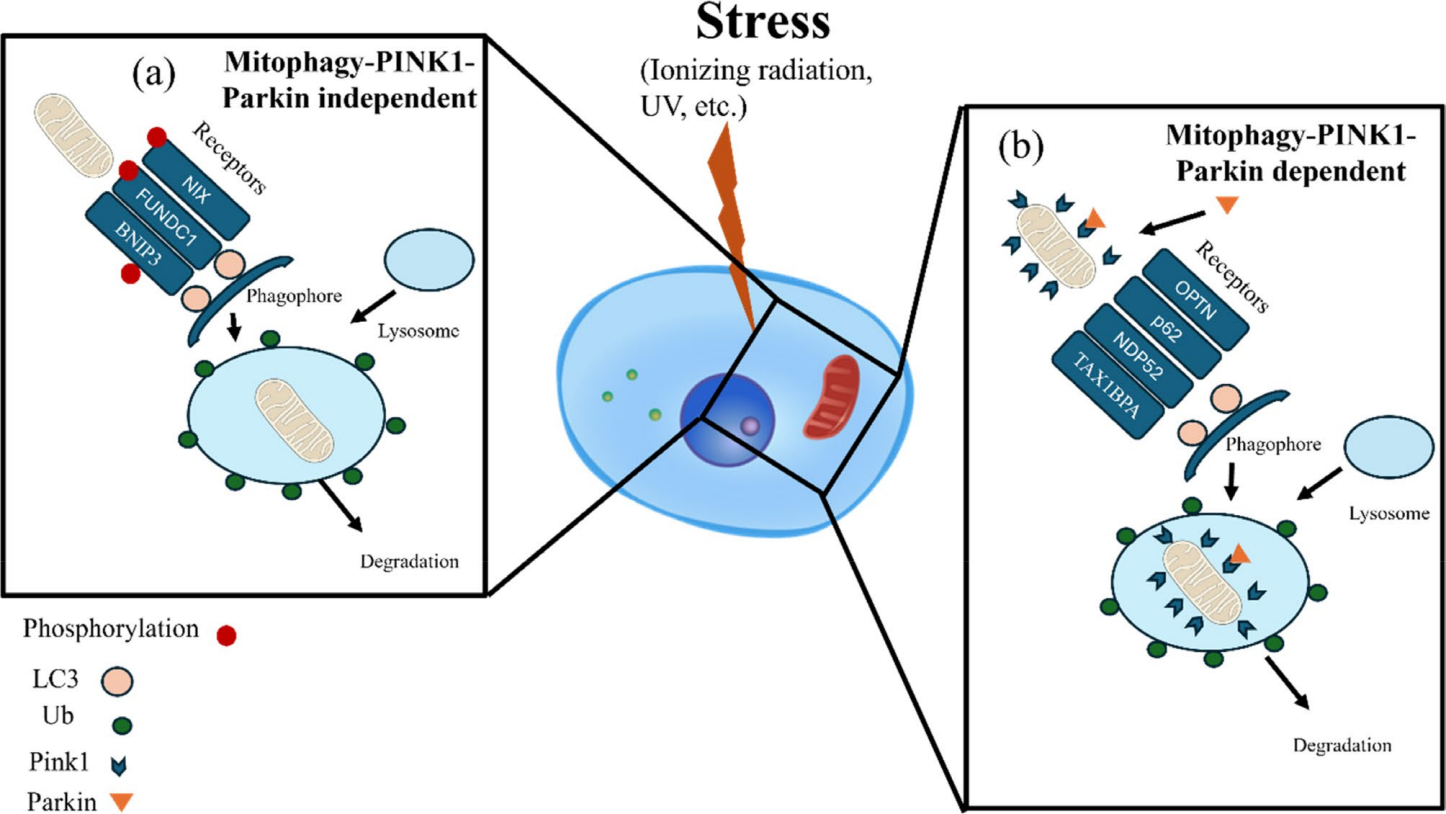
Mitochondrial dynamics and quality control mechanisms. (a) Mitophagy PINK1/Parkin‐independent pathway, where mitophagy receptors such as NIX, FUNDC1, and BNIP3 facilitate mitochondrial degradation by directly interacting with LC3 on the forming autophagosome, leading to lysosomal degradation. Phosphorylation events regulate receptor activation. (b) Mitophagy‐PINK1‐Parkin dependent. Mitophagy selectively removes damaged mt to maintain mt quality. Upon mt damage, PTEN‐induced kinase 1 (Pink1) accumulates on the outer membrane, recruiting E3 ubiquitin ligase Parkin, which ubiquitinates mt surface proteins. These ubiquitinated mt are recognized by autophagic receptors, sequestered into phagophores, and enclosed in autophagosomes. Fusion with lysosomes enables enzymatic degradation and recycling of mt components, preserving cellular homeostasis.

Mt biogenesis and quality control are intricately coordinated processes essential for maintaining functional mt and ensuring cellular energy demands are met. These processes rely on the interplay between nuclear and mt genomes, involving transcriptional regulation, protein import systems, and quality control mechanisms that safeguard mt integrity (Xu et al. 2025). Mt fission and fusion are also critical for maintaining mt homeostasis by regulating mtDNA stability, mitophagy, and apoptosis. More recently, nuclear epigenetic modifications have been shown to play important fundamental roles in regulating mt biogenesis, particularly through DNA methylation of key metabolic gene promoters, such as those involved in the TCA cycle (Figure 2).

### 2.1 Mitochondrial Biogenesis and Quality Control

Given the mitochondria's central role in cellular homeostasis, disruptions in mt biogenesis pathways contribute to metabolic disorders, aging, and various mt diseases (Xu et al. 2025). The regulation of mt biogenesis is primarily driven by nuclear‐encoded transcriptional factors that modulate the expression of mt proteins required for oxidative phosphorylation (OXPHOS), mtDNA replication, and metabolic adaptation. Among these regulators, peroxisome proliferator‐activated receptor gamma coactivator 1‐alpha (PGC‐1α) plays a pivotal role as a transcriptional coactivator, enhancing the expression of nuclear‐encoded mt genes (Li et al. 2022). PGC‐1α (Peroxisome proliferator‐activated receptor gamma coactivator 1‐alpha) exerts its effects by interacting with nuclear respiratory factors (NRF1 and NRF2), which directly activate genes encoding components of the electron transport chain (ETC), mt ribosomal proteins, and mt chaperones (He et al. 2020; Zhang et al. 2024a; Zhang et al. 2024b). Additionally, mt TFAM, a downstream effector of PGC‐1α, binds directly to mtDNA, regulating its transcription and replication. Dysregulation of these transcriptional pathways is associated with mt dysfunction, contributing to neurodegenerative diseases and metabolic syndromes.

Mt biogenesis requires precise coordination between nuclear and mt genomes to maintain the balance of nuclear‐encoded proteins, which are essential for mt function through proper import and integration into mt compartments. This coordination is facilitated by retrograde signaling pathways, enabling mt to communicate their functional status to the nucleus and modulate gene expression in response to bioenergetic needs (Sharma et al. 2021). Mt retrograde signaling involves stress‐responsive transcription factors such as activating transcription factor 4 (ATF4) and nuclear factor erythroid 2‐related factor 2 (Nrf2), which regulate antioxidant defense mechanisms and mt metabolism (Sarcinelli et al. 2020; Wortel et al. 2017). Since mt proteins are predominantly synthesized in the cytoplasm, efficient import and sorting systems are required for their proper localization. The translocation of mt proteins is mediated by two major complexes: the translocase of the outer membrane (TOM) and the translocase of the inner membrane (TIM) systems. The TOM complex (TOM20, TOM40, TOM70) recognizes precursor proteins and facilitates their translocation across the outer mt membrane. Proteins destined for the inner membrane or matrix are further processed by the TIM23 and TIM22 complexes, ensuring their proper integration (Boas et al. 2022). To prevent protein misfolding and aggregation, mt chaperones such as heat shock protein 70 (mtHSP70) and heat shock protein 60 (HSP60) assist in folding newly imported proteins, maintaining mt proteostasis (Singh, Shin, et al. 2024). Dysfunction in these import and folding pathways leads to mt proteotoxic stress, which can ultimately trigger apoptosis and cellular dysfunction.

Maintaining mt proteostasis requires robust quality control mechanisms that detect and eliminate misfolded or damaged proteins. One of the key adaptive responses to mt stress is the mt unfolded protein response (UPRmt), which is activated to restore proteostasis when misfolded proteins accumulate (Yamano et al. 2024). This response is mediated by transcription factors such as ATF5 and CHOP, which upregulate mt chaperones and proteases to mitigate proteotoxic stress (Cilleros‐Holgado et al. 2023). Additionally, the ClpXP protease system–a complex of ClpX and ClpP–functions as part of the mt degradation machinery, selectively removes damaged proteins to prevent their accumulation. Defects in these quality control systems contribute to mt dysfunction and are implicated in aging, neurodegenerative diseases, and metabolic disorders. Enhancing these pathways has therefore emerged as a potential therapeutic strategy to improve mt resilience and maintain cellular homeostasis (Hipp and Hartl 2024). A crucial layer of mt regulation is mediated by sirtuins, a family of NAD^+^‐dependent deacetylases that influence mt function through epigenetic and post‐translational modifications. SIRT1, a nuclear sirtuin, enhances mt biogenesis by deacetylating and activating PGC‐1α, leading to increased expression of nuclear‐encoded mt genes (Pérez et al. 2024; Zhang, Shrivastava, et al. 2024). Within mt, SIRT3 regulates metabolic processes and oxidative stress responses by deacetylating key enzymes involved in the tricarboxylic acid (TCA) cycle, fatty acid oxidation, and the ETC. The interplay between sirtuins and mt stress responses is essential for metabolic adaptation and longevity (Pérez et al. 2024). Notably, age‐related declines in sirtuin activity are associated with mt dysfunction, underscoring the significance of sirtuin‐mediated regulation in aging and disease progression (Samoilova et al. 2024). Together, mt biogenesis and quality control represent highly integrated processes that sustain cellular energy balance and proteostasis. The transcriptional regulation of mt biogenesis, protein import systems, and mt quality control mechanisms—such as the UPRmt and ClpXP protease system—work in concert to maintain mt functionality. Emerging evidence further highlights the role of mt epigenetics and sirtuin‐mediated signaling in fine‐tuning these processes.

### 2.2 The Role of Fusion and Fission in mtDNA Maintenance

The coordinated balance of fusion and fission allows cells to eliminate defective mtDNA copies, ensuring mt quality control. When mtDNA damage increases, mt activate retrograde signaling pathways, leading to elevated ROS levels or an increased AMP/ATP ratio. These stress signals trigger a nuclear response, upregulating the expression of nuclear‐encoded mt proteins that regulate mt fission, fusion, and mitophagy (Al Ojaimi et al. 2022). This coordinated response preserves mtDNA integrity and facilitates the removal of damaged mt, maintaining overall mt function (Tábara et al. 2024).

Fusion enables the mixing of mt contents, including mtDNA, proteins, and lipids, which is essential for maintaining mt function and genetic stability (Chan 2020). By fusing, mt dilute damaged components with healthy ones from other mt, effectively reducing localized defects. This process ensures even distribution of genetic material and minimizes the impact of mutations or damage. Key proteins involved in mt fusion include Mitofusin 1 (Mfn1), Mitofusin 2 (Mfn2), which facilitate outer membrane fusion, and Optic Atrophy 1 (Opa1), which mediates inner membrane fusion (Youle and van der Bliek 2012). Fusion promotes genetic diversity and functional complementation, allowing healthy mt to compensate for deficiencies in damaged ones, thereby enhancing overall mt function (Adebayo et al. 2021; Westermann 2002). Furthermore, fusion maintains the mt membrane potential (Δψm), which is essential for ATP production through oxidative phosphorylation. By distributing the electrochemical gradient evenly across fused mt, fusion supports efficient energy production and prevents localized electrical failures (Adebayo et al. 2021; Sharma and Sampath 2019).

Conversely, mt fission is essential for segregating and removing damaged mt. Fission sustains mt integrity by isolating defective parts of mt, which are then targeted for removal through mitophagy. This selective degradation prevents the accumulation of dysfunctional mt that could produce harmful level of ROS and impair cellular health (Ni et al. 2015; Yu et al. 2023). During fission, mt divide into smaller units, which can be targeted for degradation via mitophagy (Westermann 2010). Overall, mt fusion, fission, and mitophagy function as interconnected processes that sustain mt homeostasis. Fusion enables functional complementation and genetic stability, while fission and mitophagy facilitate the removal of dysfunctional mt, preventing the propagation of mtDNA mutations. The precise coordination of these processes is essential for maintaining mt function, protecting cells from oxidative damage, and ensuring efficient energy production.

### 2.3 Epigenetic Regulation of mtDNA Maintenance

Epigenetic modifications influence the transcriptional activity of nuclear‐encoded genes that govern mt function, energy production, and oxidative metabolism. Hypermethylation of TCA cycle gene promoters has been associated with decreased metabolic activity and reduced ATP production, particularly in aging and metabolic disorders (Mishra et al. 2022). This epigenetic silencing limits the availability of metabolic intermediates essential for mt biogenesis, leading to impaired energy homeostasis and increased susceptibility to mt dysfunction (Sharma et al. 2021). Conversely, demethylation of these promoters enhances gene expression, promoting TCA cycle activity, OXPHOS, and mt adaptation to metabolic demands. Additionally, non‐coding RNAs (ncRNAs), such as microRNAs (miRNAs) and long non‐coding RNAs (lncRNAs), serve as epigenetic regulators of nuclear‐encoded mt genes. Specific miRNAs can suppress mt biogenesis by targeting transcription factors such as PGC‐1α, reducing their expression. In contrast, lncRNAs can act as scaffolds for chromatin‐modifying complexes, influencing histone modifications and regulating the transcription of metabolic genes (Donato et al. 2024; Li and Kang 2024). These regulatory mechanisms allow mt biogenesis to respond dynamically to environmental and physiological stimuli, such as nutrient availability, stress, and exercise. Beyond direct effects on metabolic genes, DNA methylation patterns also regulate transcription factors such as PGC‐1α, a master regulator of mt biogenesis (Donato et al. 2024). Investigating how DNA methylation at TCA cycle gene promoters, histone modifications, and ncRNAs collectively regulate nuclear‐encoded mt genes offers valuable insights into the epigenetic control of cellular metabolism and may reveal therapeutic targets for metabolic and age‐related diseases.

Mt‐derived metabolites, particularly acetyl‐CoA and NAD+, are crucial mediators of histone modifications, linking mt metabolism to nuclear gene expression. Acetyl‐CoA, a central intermediate in the TCA cycle, acts as the primary substrate for histone acetyltransferases (HATs), promoting histone acetylation and transcriptional activation. Mt function directly influences acetyl‐CoA availability, as it is generated through ATP‐citrate lyase (ACLY) activity and transported to the nucleus via the citrate transporter (SLC25A1) (Passos et al. 2024; Russo et al. 2023). NAD+, a vital coenzyme in redox reactions, serves as a substrate for sirtuins, which facilitate histone deacetylation, leading to transcriptional repression in response to metabolic stress. Through NAD+‐dependent deacetylation, sirtuins regulate nuclear genes involved in mt biogenesis, defence against oxidative stress, and longevity pathways. The dynamic interplay between histone acetylation and deacetylation, dictated by acetyl‐CoA and NAD+ levels, underscores mt's role as a metabolic sensor, adapting nuclear gene expression to cellular energy needs (Huang et al. 2023). Dysregulation of these metabolite‐driven epigenetic modifications has been implicated in aging, cancer, and metabolic disorders, highlighting the necessity for continued research on how mt metabolism influences chromatin remodeling and transcriptional control.

TFAM is a key regulator of mtDNA integrity, chromatin organization, and transcriptional activity, playing a critical role in mt homeostasis. As an HMG (high‐mobility group) box protein, TFAM binds to mtDNA and compacts it into nucleoids, protecting it from oxidative damage while facilitating replication (Urrutia et al. 2024). This nucleoid structure is essential for mtDNA stability, as it governs access to replication and repair enzymes such as uracil‐DNA glycosylase 1 (UNG1) and AP endonuclease I (APE1), which prevent the accumulation of mutations (Urrutia et al. 2024). Beyond its structural role, TFAM is indispensable for mt transcription, as it recruits mt RNA polymerase and other transcription factors necessary for the expression of OXPHOS (Bharathi et al. 2025). TFAM levels closely correlate with mtDNA copy number, where overexpression promotes mt biogenesis, while depletion results in severe mt dysfunction. Additionally, TFAM is closely linked to nuclear‐mt communication, coordinating the expression of mt‐encoded genes with nuclear‐encoded mt proteins to maintain a balanced mt proteome (Zhang, Lee, et al. 2024). TFAM dysregulation has been associated with aging, metabolic disorders, and neurodegenerative diseases, underscoring its critical role in maintaining mt homeostasis and its potential as a therapeutic target for mitigating mt dysfunction.

## 3 Mitophagy in mtDNA Maintenance

Mitophagy is a selective autophagic process responsible for the degradation of dysfunctional mt, playing a crucial role in mt quality control and homeostasis (Chen et al. 2020) (Figure 2b). The most well‐characterized pathway involved in mitophagy is the PINK1/Parkin signaling cascade, which relies on the mt membrane kinase PTEN‐induced kinase 1 (PINK1) and the cytosolic E3 ubiquitin ligase Parkin (Wang et al. 2021). However, there are many emerging studies revealing alternative pathways as well (Delgado and Shoemaker 2024).

### 3.1 PINK1/Parkin‐Dependent Mitophagy

Under normal conditions, PINK1 is continuously imported into healthy mt and degraded by mt proteases. However, when mt experience a loss of membrane potential—a hallmark of mt dysfunction—PINK1 accumulates on the outer mt membrane. This accumulation promotes PINK1 autophosphorylation, leading to the recruitment and activation of Parkin, which subsequently ubiquitinates mt outer membrane proteins. These ubiquitination events serve as a signal for the recruitment of autophagy adaptors such as SQSTM1/p62, which facilitate the interaction between ubiquitinated mt and microtubule‐associated protein 1 light chain 3 (LC3)‐positive autophagosomes, leading to their degradation via the lysosomal pathway (Wang et al. 2021). A key function of mitophagy is the elimination of mt containing damaged or mutated mtDNA. Since mt with mtDNA mutations often exhibit depolarization of the mt membrane, they fail to reintegrate into the mt network after fission and are instead targeted for degradation (Twig and Shirihai 2011; Uoselis et al. 2023). In human cell line experiments overexpression of Parkin enhances mt clearance, reducing the burden of deleterious mtDNA mutations. Impairments in this process lead to the accumulation of pathogenic mtDNA mutations‐particularly in neurodegenerative diseases such as Parkinson's disease‐ and drive a metabolic shift from OXPHOS to glycolysis, a hallmark of mt dysfunction (Herst et al. 2017). Defects in mitophagy have been linked to the accumulation of pathogenic mtDNA mutations, particularly in neurodegenerative diseases such as Parkinson's disease (Corti et al. 2020; Sliter et al. 2018). In experimental models, Parkin knockout (Parkin−/−) and PINK1 knockout (PINK1−/−) mice exhibit progressive mt dysfunction, leading to the loss of dopaminergic neurons, a key feature of Parkinson's disease (Matheoud et al. 2019; Sliter et al. 2018). Similarly, in Parkin knockout mouse models, an increased mtDNA mutation rate is observed in somatic cells, further supporting the role of Parkin in eliminating defective mt (Suen et al. 2010). Interestingly, the ability of mitophagy to selectively target mutated mtDNA remains a subject of debate. Some studies suggest that Parkin‐mediated mitophagy does not always eliminate mtDNA mutations efficiently. For example, study using Drosophila models show that loss of Parkin does not significantly affect the frequency of mtDNA mutations impacting COX1 protein expression, suggesting that Parkin‐independent mechanisms also contribute to mtDNA quality control (Ma et al. 2014). Additionally, when Parkin knockout mice are crossed with mtDNA mutant mice, no significant changes in total mtDNA mutational load are observed (Shen et al. 2015). Additionally, a recent study suggests that mitophagy may not be required for the elimination of mtDNA damage under certain conditions. For instance, in experiments where mt‐targeted restriction enzymes are used to induce double‐strand breaks in mtDNA, mutant mtDNA is cleared independently of mitophagy (Moretton et al. 2017). These findings raise questions about whether mitophagy directly removes mutated mtDNA or if additional pathways are involved.

Mt mutations can evade mitophagy in certain cases, allowing dysfunctional mt to persist. In mouse models deficient in mt DNA polymerase gamma (Pol‐γ), mtDNA mutations are linked to increased hepatic mitophagy, suggesting that the accumulation of mtDNA defects may act as a trigger for mitophagy (Sun et al. 2015). Mitophagy is typically initiated by mt membrane depolarization and mTOR inhibition, activating pathways such as BCL2 and PINK1/Parkin to eliminate damaged mt (Marin et al. 2013; Yang et al. 2019). Notably, mitophagy activation relies on low membrane potential, which serves as a signal for mt dysfunction. This process is crucial for maintaining cellular homeostasis by preventing the accumulation of dysfunctional mt, which can lead to increased oxidative stress and contribute to disease.

### 3.2 Alternative Mitophagy Pathways

While the PINK1/PARKIN‐mediated pathway has been extensively characterized, several alternative mitophagy pathways function independently of this ubiquitin‐dependent mechanism. These alternative pathways include BNIP3/Nix‐mediated mitophagy, PI3K‐dependent autophagy under nutrient deprivation, and lipid‐driven mitophagy initiated by cardiolipin externalization. Each of these mechanisms provides a distinct means of regulating mt turnover under specific physiological and stress conditions (Dombi et al. 2018; Gao et al. 2020). The BNIP3 (Bcl‐2/adenovirus E1B 19 kDa protein‐interacting protein 3) and Nix (also known as BNIP3L) pathway represent a receptor‐mediated form of mitophagy that does not require ubiquitination. BNIP3 and Nix are hypoxia‐inducible proteins belonging to the Bcl‐2 family, primarily involved in mt clearance under low‐oxygen conditions and during erythrocyte maturation (Niemi and Friedman 2024). These proteins localize to the outer mt membrane, where their LC3‐interacting regions (LIR motifs) facilitate direct recruitment of autophagosomes, ensuring the targeted degradation of mt. BNIP3 and Nix function by disrupting mt membrane potential (ΔΨm), leading to mt depolarization and subsequent sequestration into autophagosomes. Additionally, BNIP3 inhibits Bcl‐2 and Bcl‐xL, promoting mt dysfunction and increasing mitophagic turnover (Wang et al. 2024). This pathway plays a crucial role in hypoxia‐adaptive responses, preventing excessive ROS accumulation and mitigating oxidative stress‐induced cellular damage.

In addition to stress‐induced mt clearance, mitophagy is regulated by phosphoinositide 3‐kinase (PI3K)‐dependent autophagy, particularly in response to nutrient deprivation. During cellular starvation, class III PI3K (Vps34) plays a central role in autophagosome formation, facilitating both bulk autophagy and mitophagy to maintain metabolic balance and sustain energy production (Metur and Klionsky 2024). Under nutrient deprivation, the suppression of the mammalian target of rapamycin complex 1 (mTORC1) activates autophagy through enhanced PI3K signaling, which subsequently stimulates ULK1/2 (Unc‐51 like autophagy activating kinase 1 and 2). ULK1/2 phosphorylates autophagy‐related proteins (ATGs), initiating the formation of autophagosomes that engulf mt based on metabolic demand rather than mt damage (Feng et al. 2024; Lee et al. 2023). This pathway operates independently of the PINK1/PARKIN system and does not require mt depolarization for degradation (Delgado and Shoemaker 2024). Instead, mt are selectively removed based on metabolic necessity, making this pathway critical for cellular adaptation to fluctuating nutrient availability. Disruptions in PI3K‐mediated mitophagy have been linked to metabolic disorders, where impaired autophagic responses contribute to energy imbalances and mt dysfunction (Zhang et al. 2022).

Beyond protein‐mediated pathways, mt lipids also play a direct role in mitophagy regulation. Cardiolipin, a mt‐specific phospholipid predominantly located in the inner mt membrane, serves as a key signal for mitophagy initiation. Under mt stress, cardiolipin becomes externalized to the outer mt membrane, where it acts as an autophagy signal by directly interacting with LC3, a core component of the autophagosomal membrane. This lipid‐based recognition mechanism provides an alternative mitophagic pathway, independent of ubiquitin signaling (Jarocki et al. 2024). The translocation of cardiolipin to the outer membrane occurs in response to mt damage, oxidative stress, and calcium overload. The enzyme phospholipid scramblase 3 (PLSCR3) mediates this process by translocating, cardiolipin to the outer mt membrane, where it becomes accessible to cytosolic LC3. This interaction facilitates the recruitment of autophagosomes to the mt, marking them for degradation. In addition to its role in mitophagy, cardiolipin is essential for maintaining mt membrane integrity and optimizing ETC function (Jarocki et al. 2024; Yoshii et al. 2024). Defective cardiolipin metabolism is linked to various diseases, particularly Barth syndrome, a genetic disorder characterized by mt dysfunction due to abnormalities in tafazzin, an enzyme responsible for cardiolipin remodeling (Jarocki et al. 2024). Thus, beyond its structural role, cardiolipin plays an essential role in mt quality control, as highlighted by these findings (Yang, Li, et al. 2024). Together, these non‐canonical pathways expand the mitophagy landscape, highlighting the cells’ ability to fine‐tune mt quality control in response to diverse stressors–offering promising avenues for therapeutic intervention in mt diseases.

### 3.3 Mechanisms of mtDNA Mutation Escape From Mitophagy

Despite the presence of quality control mechanisms, certain mtDNA mutations can evade mitophagy by inducing mt hyperpolarization, which prevents their degradation (Knorre 2020). Additionally, dysfunctional mt may escape clearance via intercellular mt transfer, allowing damaged organelles‐and their pathological mtDNA variants‐to migrate into neighboring cells and contribute to disease progression (Dickson‐Murray et al. 2021).

Understanding how certain mtDNA mutations bypass mitophagy through hyperpolarization or intercellular transfer is essential for developing therapeutic strategies aimed at enhancing mt quality control in neurodegenerative and metabolic disorders.

## 4 Nuclear‐Mt Interactions in mtDNA Repair

MtDNA has a higher mutation rate than nuclear DNA, a phenomenon initially attributed to its proximity to the ETC, which exposes it to ROS. These byproducts of mt respiration, including superoxide anions, hydroxyl radicals, and hydrogen peroxide, can interact with DNA bases such as guanine and thymine, leading to oxidative modifications like 8‐oxo‐7,8‐dihydroguanine (8‐oxo‐dG) and 5,6‐dihydroxy‐5,6‐dihydrothymine. However, recent studies suggest that superoxide anions are weak DNA modifiers, while hydrogen peroxide rapidly diffuses out of the mt, minimizing their direct damage (Halliwell and Aruoma 1991; Lesko et al. 1980). The limited impact of ROS on mtDNA mutations is further supported by findings that only a small fraction of cellular oxygen is converted to superoxide, which is rapidly neutralized by mt superoxide dismutases in the matrix and intermembrane space. In addition, despite the ETC's proximity to mtDNA, its exposure to ROS is mitigated by its packaging within nucleoids, where mt TFAM plays a protective role by shielding it from oxidative stress (Bharathi et al. 2025; Stenberg et al. 2022). A study by Kauppila et al. showed that mt point mutation rates remained unchanged in wild‐type mice and those lacking DNA glycosylases such as OGG1 and MYH, as well as in SOD2 knockout mice (Kauppila et al. 2018). This challenges the idea that oxidative damage is the primary driver of mtDNA mutations. Instead, the accuracy of mtDNA replication, largely influenced by DNA polymerase gamma (Polγ), may play a critical role in preventing mutations. Unlike most polymerases, Polγ exhibits high fidelity, particularly when encountering 8‐oxo‐dG lesions. Rather than incorporating incorrect nucleotides, Polγ preferentially inserts cytosine, thereby reducing the frequency of G → T transversion mutations (Brieba et al. 2004). Research has shown that spontaneous deamination, rather than oxidative damage, is the primary cause of mtDNA point mutations. This also explains why the heavy (H)‐strand of mtDNA is enriched in guanine the accumulation of G → A and T → C transitions predominantly affects the light (L)‐strand over time (Kennedy et al. 2013; Stojkovič et al. 2016). In addition to oxidative damage, mtDNA is susceptible to alkylation, including O6‐methylguanine formation and other bulky DNA adducts that also contribute to its mutational burden.

Recent research has revealed that mt have a more extensive DNA repair system than previously recognized (Druzhyna et al. 2008). While mtDNA repair still relies on nuclear‐encoded proteins, numerous mt repair enzymes have been identified in recent years. Base excision repair (BER) remains the primary pathway responsible for correcting mtDNA damage, though additional repair mechanisms have been identified (Zinovkina 2018). Mt BER operates similarly to its nuclear counterpart, though not all nuclear BER proteins are present in the mt Of the 12 known mammalian glycosylases involved in recognizing and excising damaged bases, seven are active in mt, including uracil‐DNA glycosylase (UDG), 8‐oxoG DNA glycosylase 1 (OGG1), and MYH DNA glycosylase (Plotz et al. 2012). The transport of these repair proteins into mt is dependent on the presence of a mt targeting signal (MTS) or the mt import assembly (MIA) pathway. Some nuclear DNA repair proteins have distinct mt isoforms or undergo alternative splicing to generate nuclear and mt variants. Mt‐specific forms of OGG1, NTH1, and MYH DNA glycosylase have also been identified, emphasizing their role in maintaining both mt and nuclear genome stability (de Souza‐Pinto et al. 2001; García‐Lepe and Bermúdez‐Cruz 2019; Takao et al. 2002). Certain proteins, such as Exo/Endonuclease G (EXOG1), appear to function exclusively in mt, indicating that the mt has evolved specialized repair mechanisms (Szymanski et al. 2017). Beyond BER, mt employ additional repair pathways, including direct reversal mechanisms, mismatch repair, double strand break repair, and nucleotide excision repair (Saki and Prakash 2017).

Despite significant advancements in identifying and characterizing repair pathways and proteins involved in mtDNA maintenance, the process by which mtDNA damage is detected and relayed to initiate appropriate repair responses remains elusive. For example, it is still unclear how signals from mtDNA lesions are transmitted to the nucleus or cytoplasm to regulate the expression of repair proteins or facilitate the translocation of existing repair enzymes into the mt.

### 4.1 Nuclear‐Encoded DNA Repair Proteins Targeting Mitochondria

The mt recruitment of nuclear‐encoded DNA repair proteins is often tightly controlled, with some responding broadly to oxidative stress and others acting in direct response to mtDNA damage. For instance, apurinic/apyrimidinic endonuclease 1 (APE1) (Frossi et al. 2002), Cockayne syndrome A and B proteins (ERCC6 and ERCC8) (Kamenisch et al. 2010; Stevnsner et al. 2008), and Rad51 (Sage and Knight 2013) are cytosolic under basal conditions but translocate to the mt following oxidative stress. In contrast, certain repair enzymes, including OGG1 and NTH1, respond specifically to mtDNA damage and translocate to the mt independent of ROS levels (Ikeda et al. 2002; Shinmura and Yokota 2001; Swartzlander et al. 2010). Notably, poly (ADP‐ribose) polymerase 1 (PARP1), a key nuclear repair regulator, is among the first proteins to be localize to mt under oxidative stress conditions. However, it remains uncertain whether the nuclear and mt forms of PARP1 are identical or distinct isoforms (Bürkle and Virág 2013; Szczesny et al. 2014). Interestingly, unlike its role in nuclear DNA repair, mt PARP1 has been observed to regulate mtDNA repair negatively. It interacts with various repair proteins involved in BER and nucleotide excision repair (NER), such as Polγ, EXOG1, TFAM, Cockayne syndrome B (CSB), aprataxin (APTX), and tyrosyl‐DNA phosphodiesterase 1 (TDP1). Additionally, PARP1 is known to associate with multiple enzymes of the ETC and Krebs cycle, impairing replication, transcription, and respiration, leading to the depletion of NAD+ and acetyl‐CoA (Fang et al. 2016). The depletion of NAD+ represents a critical regulatory axis in mt genome stability, linking nuclear‐mitochondrial repair processes with metabolic stress responses. NAD+ is essential for mitochondrial function, serving as a cofactor for key enzymes involved in DNA repair, redox balance, and sirtuin‐dependent stress adaptation. PARP1‐mediated NAD+ depletion impairs mt BER efficiency by limiting the availability of NAD+‐dependent enzymes such as SIRT1 and SIRT3, which regulate mtDNA repair and oxidative stress responses. SIRT1 modulates the nuclear transcriptional response to mitochondrial stress, while SIRT3 functions within mt to maintain genome stability by deacetylating DNA repair factors such as OGG1 and APE1 (Bai et al. 2011; Fan et al. 2018). Moreover, NAD+ availability influences the recruitment of nuclear‐encoded repair proteins to mt under stress conditions. The nuclear‐mitochondrial shuttling of key repair enzymes, including APE1, TFAM, and EXOG1, is regulated in part by fluctuations in NAD+ levels, affecting their ability to sustain mtDNA repair under conditions of oxidative stress and metabolic perturbations. Low NAD+ levels impair this process, increasing susceptibility to mtDNA mutations and mitochondrial dysfunction (Luo and Kraus 2012). Conversely, interventions that restore NAD+ levels, such as nicotinamide riboside supplementation, have been shown to enhance mtDNA stability and promote mitochondrial quality control (Verdin 2015). Another protein that translocates to the mt under oxidative stress conditions is p53, which plays an essential role in BER by interacting with single‐stranded DNA binding protein (SSB), Polγ, OGG1, APE1, and TFAM. Given the central role of NAD+ in both mitochondrial and nuclear repair pathways, the interplay between sirtuins, PARP1, and mitochondrial repair enzymes constitutes a critical mechanism for ensuring mtDNA integrity and promoting cellular resilience under metabolic stress.

### 4.2 Regulation of Mt Genome Integrity by Nuclear Factors

The integrity of the mt genome is maintained through the coordinated actions of nuclear‐encoded factors, which regulate mtDNA packaging, repair, and stress responses. Among these regulators, TFAM plays a fundamental role in organizing and stabilizing mtDNA, ensuring proper maintenance of the mt genome. It is also believed to serve as an early responder to mtDNA damage (Park et al. 2016). TFAM appears to temporarily suppress replication, transcription, and repair activities upon sensing mtDNA damage, creating a window for repair proteins to access the genome and initiate appropriate responses (Kang and Hamasaki 2005; Szczesny et al. 2014). Additionally, crosstalk between the DNA damage response (DDR) and mt stress signaling is essential for preserving mt integrity, with sirtuins, particularly SIRT1 and SIRT3, serving as key modulators of these processes (Bosch‐Presegué and Vaquero 2014; Rasti et al. 2023; Weng et al. 2020). This nuclear‐mt interplay is crucial for preventing genomic instability, optimizing mt function, and sustaining cellular homeostasis. TFAM is a nuclear‐encoded high‐mobility group (HMG) box protein that serves as a primary regulator of mtDNA packaging, transcription, and stability. It binds to mtDNA and facilitates its compaction into nucleoids, protecting it from degradation and oxidative damage. This structural organization is crucial for mt genome maintenance, as it ensures proper replication and transcription while preventing excessive strand breaks (Bharathi et al. 2025; Weng et al. 2020; Wu et al. 2019). Beyond its role in packaging, TFAM exhibits dual functionality by bending DNA to initiate transcription while also stabilizing mtDNA. Additionally, TFAM possesses dRp lyase activity, which assists in removing toxic DNA repair intermediates, preventing mt genome fragmentation and dysfunction (Zhao et al. 2024). Dysregulation of TFAM leads to mtDNA instability, impaired ETC function, and increased susceptibility to oxidative stress‐related diseases such as neurodegeneration and metabolic disorders.

The interplay between DDR and mt stress signaling is a critical mechanism by which nuclear and mt genomes coordinate genomic stability and repair responses. DDR, a well‐established pathway for detecting and repairing nuclear DNA damage, also influences mtDNA integrity by regulating mt stress responses (Ma and Zhou 2025). TFAM acts as a mediator between these pathways, modulating the activity of DNA repair enzymes such as APE1 and UNG1 to address mtDNA lesions (Urrutia et al. 2024). When mt stress occurs due to oxidative damage or replication errors, nuclear DDR pathways communicate with mt to enhance repair capacity, regulate mitophagy, and prevent the accumulation of defective mtDNA (King et al. 2024). Disruption of this crosstalk leads to mt dysfunction, aging‐related genomic instability, and increased susceptibility to diseases. As critical regulators of mt function, SIRT1 and SIRT3, help preserve genomic stability by coordinating repair mechanisms with mt homeostasis. SIRT1, primarily localized in the nucleus, enhances mt biogenesis by deacetylating PGC‐1α, a master regulator of nuclear‐encoded mt genes. It also influences DDR signaling, ensuring that mt repair pathways are efficiently activated in response to stress. SIRT3, localized within the mt, plays a direct role in mtDNA repair and genome maintenance by regulating the activity of superoxide dismutase 2 (SOD2) and other antioxidant enzymes, thereby reducing oxidative damage to mtDNA (Zhang et al. 2023). Furthermore, SIRT3 deacetylates key mt enzymes involved in DNA repair and energy metabolism, promoting cellular adaptation to metabolic stress (Bharathi et al. 2025). Understanding these nuclear‐driven regulatory mechanisms provides critical insights into aging, metabolic disorders, and neurodegenerative diseases, highlighting potential therapeutic strategies to preserve mt genome integrity in pathological conditions.

## 5 Integrated Stress Response in mtDNA Crosstalk and Genomic Stability

The Integrated Stress Response (ISR**)** is crucial for coordinating communication between nucleus and mt, integrating stress signals from the mt, ER, and cytosol to maintain cellular homeostasis. One critical ISR pathway is the PERK‐ATF4 axis, which links mitochondrial and ER stress responses. Under conditions of ER stress, Protein Kinase R‐like ER Kinase (PERK) phosphorylates eIF2α, leading to selective translation of Activating Transcription Factor 4 (ATF4) (Ronayne and Latorre‐Muro 2024). While originally characterized as part of the Unfolded Protein Response (UPR**)**, ATF4 activation also occurs independently via other ISR kinases (such as General control nonderepressible 2 or GCN2; Protein kinase R or PKR and Heme‐regulated inhibitor or HRI) in response to mitochondrial dysfunction, oxidative stress, amino acid deprivation, and proteotoxic stress. ATF4 regulates genes involved in mitochondrial homeostasis, antioxidant defense, amino acid metabolism, and autophagy, playing a crucial role in adapting to mitochondrial stress. Additionally, mitochondrial stress responses include the mitochondrial unfolded protein response (UPRmt) discussed earlier and the regulation of mt‐derived peptides (MDPs), both of which influence nuclear gene expression. These pathways collectively ensure mitochondrial quality control, metabolic adaptation, and cell survival under stress conditions (Savu and Moisoi 2022).

### 5.1 The PERK‐ATF4 Pathway

While the PERK‐ATF4 pathway is well‐known for its role in ER stress, it also coordinates responses to a variety of other stressors, including mt dysfunction, by regulating adaptive mechanisms such as antioxidant defense and redox balance (Anderson and Haynes 2020). PERK is a transmembrane sensor that detects ER stress and initiates the UPR to restore protein homeostasis. Under stress conditions, PERK undergoes autophosphorylation, leading to the phosphorylation of eukaryotic initiation factor 2 alpha (eIF2α), which transiently suppresses global protein synthesis while selectively enhancing the translation of ATF4 (Qureshi et al. 2024). ATF4 functions as a key transcription factor that regulates a network of genes involved in oxidative stress responses, amino acid metabolism, redox balance, and apoptosis. Specifically, ATF4 upregulates genes encoding antioxidant proteins, chaperones, and enzymes that mitigate ER stress, thereby promoting cellular survival. However, prolonged ATF4 activation can lead to the induction of apoptosis through the transcriptional upregulation of pro‐apoptotic factors such as CHOP (Qureshi et al. 2024; Somasundaram et al. 2024). Recent findings highlight a novel regulatory mechanism of the PERK‐ATF4 pathway through redox modulation. Polysulfides and persulfides have been shown to activate PERK through persulfidation, promoting its oligomerization and subsequent eIF2α phosphorylation. This activation enhances ATF4 nuclear accumulation, triggering the expression of cytoprotective proteins such as Sestrin2 (Koike et al. 2025). Sestrin2, regulated by ATF4, plays a vital role in oxidative stress defense and metabolic adaptation (Koike et al. 2025). In neuronal cells, Sestrin2 induction via the PERK‐ATF4 axis has been demonstrated to protect against methylglyoxal toxicity, a process linked to neurodegenerative diseases (Koike et al. 2025). These findings underscore the dual role of the PERK‐ATF4 pathway, which balances cellular survival and apoptosis depending on the intensity and duration of stress exposure. The physiological significance of the PERK‐ATF4‐mediated response extends beyond ER stress adaptation, influencing mt function, metabolic reprogramming, and immune responses. By regulating amino acid metabolism, autophagy, and antioxidant pathways, ATF4 supports cellular homeostasis under stress conditions. However, dysregulation of this pathway has been implicated in neurodegenerative disorders, cancer, and metabolic diseases. Understanding the interplay between redox signaling and PERK‐ATF4 activation may provide insights into therapeutic interventions targeting ER stress‐related pathologies.

While PERK is a major regulator of ATF4 activation under ER stress, double‐stranded RNA‐dependent protein kinase (PKR) serves as an alternative eIF2α kinase, activating ATF4 under stress conditions (Tian et al. 2021). PKR is primarily known for its role in the innate immune responses, as it is activated by viral double‐stranded RNA (dsRNA) and cellular stress signals, including oxidative stress and nutrient deprivation (Rajesh et al. 2016). Similar to PERK, PKR phosphorylates eIF2α upon activation, promoting the translation of ATF4 under conditions that may not involve ER stress. Unlike the PERK‐ATF4 axis, which primarily responds to ER protein misfolding, the PKR‐ATF4 pathway is more broadly involved in cellular stress responses, including mt dysfunction, inflammatory signaling, and metabolic adaptation (Darini et al. 2019). PKR‐mediated activation of ATF4 plays a critical role in mt‐nuclear communication, as it modulates oxidative metabolism and promotes stress‐adaptive transcriptional programs that influence mt biogenesis and quality control. Additionally, PKR activation plays a key role in immune surveillance, linking cellular stress pathways with antiviral defense mechanisms through its ability to regulate inflammatory cytokine production and apoptosis (Zappa et al. 2022). The distinct activation mechanisms of the PERK‐ATF4 and PKR‐ATF4 pathways highlight their complementary roles in cellular stress adaptation, ensuring that cells can respond efficiently to both ER stress and broader cytoplasmic stress signals.

### 5.2 The Unfolded Protein Response (UPR)

The UPR is a highly conserved cellular stress response pathway activated in response to the accumulation of misfolded or unfolded proteins within the endoplasmic reticulum (ER), a condition known as ER stress. Its primary function is to restore proteostasis within the ER by enhancing the protein‐folding capacity, degrading misfolded proteins, and reducing the overall protein load. Failure to resolve ER stress can lead to cellular dysfunction and apoptosis, underscoring the critical role of the UPR in maintaining cellular homeostasis (Di Mattia et al. 2025). The UPR is mediated by three key sensor proteins: PERK (PKR‐like ER kinase), IRE1 (inositol‐requiring enzyme 1), and ATF6 (activating transcription factor 6), each initiating distinct signaling cascades that collectively mitigate ER stress and restore cellular equilibrium (Prasad 2025).

Upon activation by ER stress, PERK dimerizes and autophosphorylates, leading to the phosphorylation of eIF2α (Wolzak et al. 2022). This phosphorylation event reduces global protein synthesis, alleviating the protein‐folding burden on the ER. However, selective translation of certain mRNAs is promoted, including transcription factor ATF4. ATF4 also promotes genomic stability by regulating genes involved in DNA repair and cell cycle control, integrating stress responses across multiple cellular compartments (Le et al. n.d.; Longo et al. 2021). Meanwhile, IRE1‐ an ER‐resident transmembrane protein with endoribonuclease activity‐oligomerizes and autophosphorylates under ER stress, leading to the unconventional splicing of XBP1 (X‐box binding protein 1) mRNA (Reuschlé et al. 2024). The spliced form of XBP1 (XBP1s) encodes a potent transcription factor that upregulates genes involved in protein quality control, including molecular chaperones, folding enzymes, and components of the ER‐associated degradation (ERAD) pathway (Le Goupil et al. 2024). These proteins enhance the ER's capacity to fold and degrade misfolded proteins, restoring ER function. XBP1s also indirectly influences mitochondrial function by modulating genes involved in lipid metabolism and mitochondrial biogenesis, highlighting the crosstalk between the UPR and mitochondrial homeostasis (Kettel and Karagöz 2024). Additionally, ATF6, an ER membrane‐bound transcription factor, is transported to the Golgi apparatus under stress conditions, where it undergoes proteolytic cleavage to release its cytosolic active form (Singh, Kaur, et al. 2024). The cleaved form of ATF6 translocates to the nucleus, where it induces the expression of genes involved in protein folding, ER‐associated degradation (ERAD), and lipid biosynthesis. These actions enhance the ER's capability to manage unfolded proteins and promote the proper folding and secretion of newly synthesized proteins. Additionally, ATF6 helps maintain ER morphology and function, further supporting cellular homeostasis (Kettel and Karagöz 2024).

The UPR is not only a critical responder to acute ER stress but also adapts to chronic stress conditions. Prolonged activation of the UPR can lead to the induction of apoptotic pathways, particularly if the stress cannot be resolved. This dual role of the UPR—as both a protector and an executor of cell fate—becomes critical when dysregulated, with its malfunction implicated in various diseases, including neurodegenerative disorders, diabetes, and cancer (Di Mattia et al. 2025). In summary, the UPR is a multifaceted signaling network that integrates stress responses across cellular compartments, ensuring proper folding, degradation, and trafficking of proteins (Lin et al. 2008; Marciniak and Ron 2006). By modulating gene expression, reducing protein synthesis, and enhancing protein quality control mechanisms, the UPR plays a pivotal role in maintaining cellular homeostasis and adapting to environmental and physiological challenges.

### 5.3 Mitochondria‐Derived Peptides (MDPs) in Nuclear‐Mt Crosstalk

Mt‐derived peptides (MDPs) are recently identified signaling molecules that mediate mt‐nuclear crosstalk and play crucial roles in cellular stress responses. These peptides are encoded by short open reading frames (sORFs) within mtDNA and regulate nuclear gene expression, metabolism, and cellular adaptation to stress. Among the most well‐studied MDPs are Mt Open Reading Frame of the 12S rRNA‐c (MOTS‐c) and Humanin, both of which participate in integrating mt function with nuclear stress responses (Li et al. 2024). MOTS‐c is a key metabolic regulator that modulates cellular stress adaptation. Under conditions of metabolic or oxidative stress, MOTS‐c is synthesized within the mt and translocates to the cytoplasm, where it interacts with nuclear transcription factors to regulate gene expression. One of its primary actions involves activating AMPK, a central energy sensor that promotes metabolic homeostasis by increasing glucose uptake, fatty acid oxidation, and mt proteostasis (Yang, Li, et al. 2024). Additionally, MOTS‐c moves to the nucleus, where it interacts with antioxidant response elements (ARE) and activates Nrf2, a transcription factor that regulates genes involved in oxidative stress responses and inflammation (Zhang, Huang, et al. 2024). Through these mechanisms, MOTS‐c helps protect against metabolic imbalances, mt dysfunction, and cellular aging.

MDPs also act as retrograde signaling molecules that influence nuclear transcriptional programs, with humanin being a prime example. Humanin is encoded by sORFs within the 16S rRNA of mtDNA and has been identified as a cytoprotective peptide involved in stress adaptation (Li et al. 2024). This peptide binds to nuclear transcription factors, such as STAT3 and FOXO3a, thereby modulating gene expression programs that promote cell survival, antioxidant responses, and mt homeostasis. Humanin exerts its effects by inhibiting pro‐apoptotic factors, including BAX and tBID, which prevent mt permeability transition and cytochrome c release, thereby suppressing apoptosis (Zhu, Li, et al. 2022; Zhu, Hu, et al. 2022). Additionally, Humanin regulates inflammation by modulating the NF‐κB pathway, leading to reduced expression of pro‐inflammatory cytokines such as TNF‐α and IL‐6 (Gong et al. 2022).

MDPs also play a role in integrating mt signaling with the ISR, a cellular defense mechanism described earlier (Sanfrancesco and Hood 2025). Recent evidence suggests that MDPs, particularly humanin and MOTS‐c, modulate ISR activation by influencing eIF2α phosphorylation and ATF4‐dependent transcriptional responses (Le et al. 2025). By acting as retrograde signaling molecules, these peptides ensure that mt stress is effectively communicated to nuclear stress response pathways, allowing cells to initiate adaptive mechanisms that enhance proteostasis, metabolism, and survival.

MDPs play a critical role in coordinating mt‐nuclear communication, particularly during stress adaptation and metabolic regulation. Peptides such as MOTS‐c and humanin function as retrograde messengers, integrating mt status with nuclear transcriptional programs to maintain cellular homeostasis (Wan et al. 2023). Their interaction with the ISR further underscores their importance in enhancing cellular resilience under stress conditions. Owing to their protective roles, MDPs have garnered interest as potential therapeutic targets for metabolic disorders, neurodegenerative diseases, and age‐related conditions (Wan et al. 2023). Elucidating their precise mechanisms of action could pave the way for novel interventions to mitigate mt dysfunction and promote cellular health.

## 6 Redox Regulation and Mt Dynamics in Cellular Stress Responses

**FIGURE 3:**
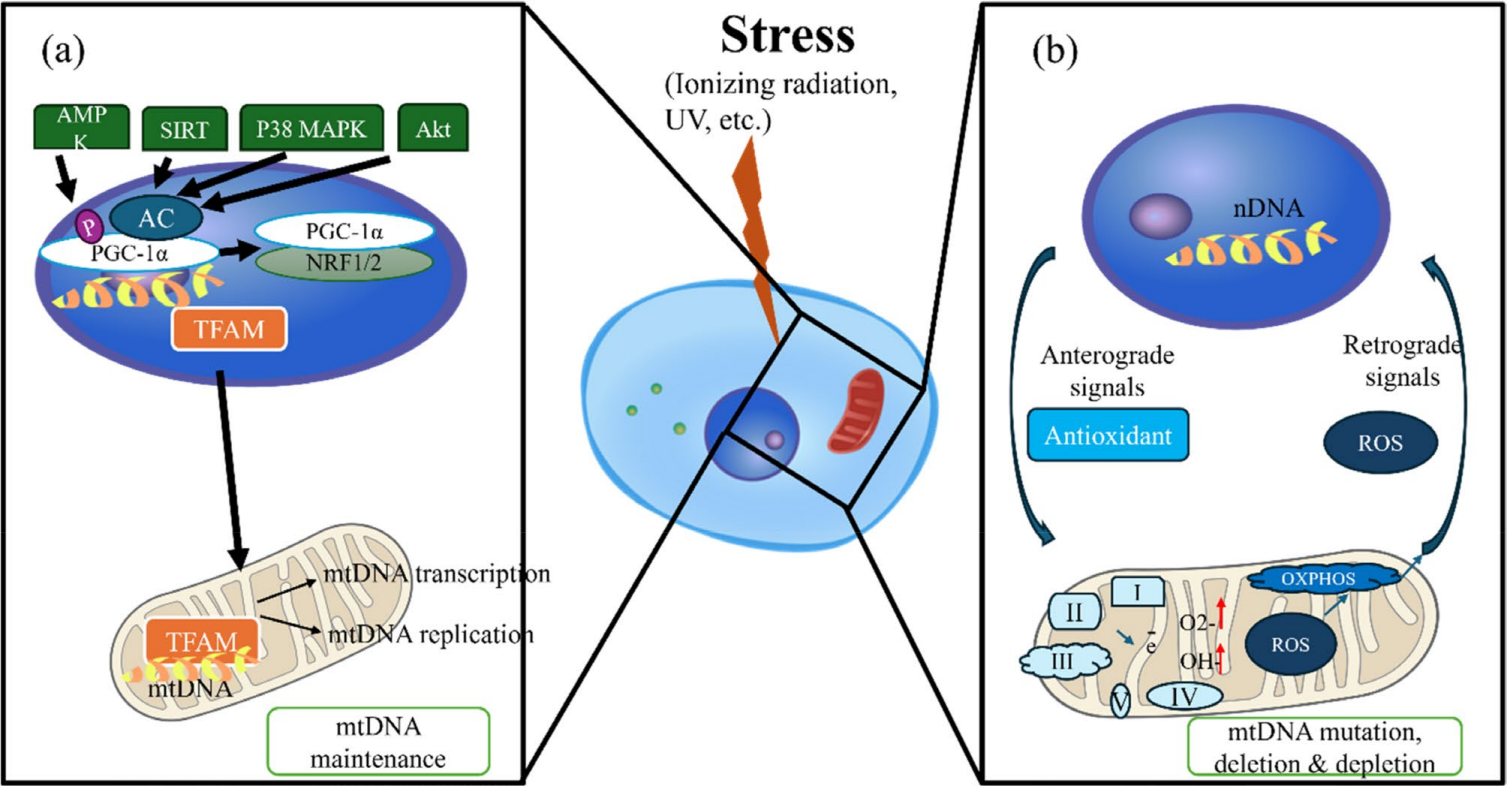
Mt DNA maintenance and mt‐nuclear communication in redox regulation. (a) mt stress activates signaling pathways, including AMPK, SIRT, P38 MAPK, and Akt, which regulate the acetylation (AC) and phosphorylation of PGC‐1α. PGC‐1α, in conjunction with NRF1/2, promotes the expression of TFAM, a key mt transcription factor. TFAM is imported into mt, where it regulates mtDNA transcription, replication, and maintenance. (b) Mt OXPHOS generates ROS, which can lead to mtDNA mutations, deletions, and loss of genome integrity. In response, mt send retrograde signals to the nucleus, modulating gene expression to mitigate damage. In turn, the nucleus initiates anterograde signals, such as the upregulation of antioxidants, to protect the mt and maintain cellular homeostasis.

Redox‐dependent signaling is essential for regulating diverse cellular processes, including differentiation, proliferation, and apoptosis. As both major sources and targets of ROS, mt are central players in maintaining redox homeostasis. Mt responses to oxidative stress are linked to nuclear regulatory programs, reflecting their evolutionary origin. The integration of anaerobic glycolysis and oxidative phosphorylation stems from the ancient endosymbiotic merger of an aerobic bacterium with a host anaerobic cell (Figure 3b). Over evolutionary time, the majority of mt genes have migrated to the nuclear genome, leaving the mt genome to encode only 13 essential subunits of respiratory chain along with essential tRNAs and rRNAs (Suliman and Piantadosi 2014; Lennicke and Cochemé 2021; Sanchis‐Gomar et al. 2014). This nuclear‐mt interdependence enables a highly coordinated redox response to metabolic and environmental stressors.

### 6.1 ROS Production and Signaling in the Mt

ROS are primarily generated in the mt, with superoxide radicals ($O_{2}^{•-}$) produced through incomplete oxygen reduction or electron leakage from the respiratory chain, particularly at complex I and complex III (Dröse and Brandt 2012). Mt ROS are byproducts of OXPHOS and play a dual role as both signaling molecules and contributors to oxidative stress. While low levels of mt ROS are essential for cellular signaling and adaptation, excessive ROS production can damage mt DNA, proteins, and lipids, leading to mt dysfunction and cellular stress (Kowalczyk et al. 2024, Lebiedzinska‐Arciszewska et al. 2025). Complex I is one of the primary sites of ROS production within the mt. It is responsible for transferring electrons from NADH to ubiquinone (CoQ), a process that contributes to the proton gradient necessary for ATP synthesis (Kowalczyk et al. 2024; Okoye et al. 2023). Under normal conditions, this electron transfer occurs efficiently, minimizing ROS formation. However, when electron flows through complex I are disrupted—such as during mt dysfunction, hypoxia, or metabolic stress—electrons can prematurely reduce oxygen, generating superoxide anions ($O_{2}^{•-}$) (Okoye et al. 2023). Reverse electron transport (RET) through complex I, which occurs when the proton‐motive force is high and electrons flow in the reverse direction from ubiquinol to NAD+, is another major mechanism of ROS generation. RET‐driven ROS production is particularly relevant in tissues with high metabolic activity, such as heart and skeletal muscle, and has been implicated in ischemia–reperfusion injury and metabolic disorders (Tabata Fukushima et al. 2024). Complex III (cytochrome bc1 complex) is another major source of mt ROS production. It transfers electrons from ubiquinol (QH_2_) to cytochrome c through the Q cycle, during which an unstable semiquinone intermediate forms at the outer quinone‐binding site (Qo site), leading to the leakage of electrons and generation of superoxide (Arantes 2025). ROS generated at complex III can be particularly damaging because they can exit the mt matrix and reach the intermembrane space, where they may influence nuclear transcriptional programs and inflammatory signaling pathways (Barnett et al. 2024). Complex III‐derived ROS play a role in the activation of NF‐κB and HIF‐1α, linking mt function with nuclear stress responses (Barnett et al. 2024).

Mt enzymes, such as superoxide dismutase, convert superoxide to hydrogen peroxide (H_2_O_2_), which prevents harmful reactions and serves as a signaling molecule (Bolisetty and Jaimes 2013). H_2_O_2_ can diffuse through membranes, regulating cellular stress responses by interacting with transcription factors, kinases, and phosphatases in a concentration‐dependent manner (Marinho et al. 2014). At higher levels, ROS can cause lipid peroxidation, generating electrophiles that modify proteins by oxidizing cysteine residues (Fischer et al. 2012; Lennicke and Cochemé 2021). This redox signaling system activates mt quality control pathways, including antioxidant defenses, metabolic reprogramming, DNA repair, protein quality control, and autophagy/mitophagy, to minimize ROS production and cellular damage. Excessive ROS levels can overwhelm these mechanisms, triggering apoptosis to limit tissue damage and prevent necrosis when stress responses fail.

Oxidative damage to mtDNA plays a pivotal role in modulating nuclear signaling pathways, thereby influencing cellular responses to stress and disease. The mt serve as critical signaling hubs, and when exposed to oxidative stress, they release ROS and damaged mtDNA into the cytoplasm. This release initiates a cascade of nuclear signaling events, including the activation of the cGAS–STING pathway, which enhances nDNA repair responses and promotes genomic stability under stress conditions (Wu et al. 2019). Consequently, oxidative mtDNA damage can trigger adaptive responses that bolster the cell's capacity to repair nuclear lesions, demonstrating a dynamic interplay between mt and nDNA in response to stress (Bhatti et al. 2021). Furthermore, the release of damaged mtDNA can activate inflammatory signaling pathways. For instance, activation of Z‐DNA binding protein 1 (ZBP1) by oxidatively modified mtDNA in epithelial cells directly links oxidative stress to inflammation. This mechanism underscores how mtDNA damage can act as a sensor of cellular stress, triggering immune responses and contributing to chronic inflammatory conditions (Szczesny et al. 2018). In addition to its role in acute stress responses, oxidative damage to mtDNA is closely associated with aging and age‐related pathologies. With advancing age, the accumulation of oxidative lesions in mtDNA correlates with a decline in mt function, which can exacerbate genomic instability and promote the development of neurodegenerative diseases (Kowalska et al. 2020). This accumulation of mtDNA mutations not only disrupts cellular energy production but also perpetuates a cycle of increased ROS production, further compromising both mt and nDNA integrity. However, this oxidative stress does not solely have deleterious effects; in certain contexts, it can activate protective signaling pathways that enhance nDNA repair and improve cellular resilience, suggesting a complex, bidirectional relationship between oxidative stress and cellular homeostasis (Kowalska et al. 2020). The interplay between oxidative damage to mtDNA and nuclear signaling pathways underscores the dual role of ROS as both damaging agents and critical signaling molecules. While excessive oxidative damage can lead to genomic instability, inflammation, and age‐related decline, controlled levels of mtDNA damage may activate compensatory mechanisms that enhance repair and promote cellular adaptation (Kowalska et al. 2020). This intricate balance is fundamental to understanding the broader implications of mt dysfunction in the context of disease and aging.

Building on the signaling role of ROS, a recent study demonstrates that mt H_2_O_2_ can directly modulate nuclear chromatin architecture, revealing a novel mechanism of mito‐nuclear communication during stress adaptation. Specifically, oxidation of histone H3.1 by mt H_2_O_2_ triggers its displacement and replacement with H3.3, a histone variant associated with active transcription. This histone exchange enables transcriptional reprogramming, allowing the cell to adapt to metabolic and environmental stressors. Rather than acting solely as harmful byproducts, ROS at controlled levels serve as regulatory molecules that tightly couple mt activity to nuclear gene expression (Rigon et al. 2021). Their influence is shaped by factors such as concentration, production kinetics, and the compartmentalized redox buffering systems that maintain cellular homeostasis.

Mt stress, arising from oxidative damage, metabolic imbalances, or impaired respiration, necessitates robust nuclear‐driven antioxidant responses to maintain cellular homeostasis. The nucleus coordinates defense mechanisms that mitigate mt stress by activating antioxidant pathways, regulating mt biogenesis, and modulating cellular metabolism. Among these responses, transcription factors such as Nrf2, FOXO3a, and NF‐κB coordinate antioxidant defense mechanisms, while a complex feedback loop involving AMPK, PGC‐1α, PPARs, sirtuins, and these same transcription factors ensures an integrated response that enhances mt function and resilience (Bakalova et al. 2022; Yang et al.; Zhou et al. 2025). Under basal conditions, Nrf2 remains sequestered in the cytoplasm by Keap1, which facilitates its ubiquitination and degradation. However, under mt stress, increased ROS, or electrophilic damage induces conformational changes in Keap1, preventing Nrf2 degradation and allowing its translocation into the nucleus (Buttari et al. 2025; Choi and Lee 2024). Once inside the nucleus, Nrf2 binds to antioxidant response elements (AREs) within the promoters of various detoxifying and antioxidant genes, leading to the transcriptional upregulation of enzymes such as superoxide dismutase (SOD1, SOD2), glutathione peroxidase (GPX), catalase, and heme oxygenase‐1 (HO‐1) (Buttari et al. 2025; Navarro and Esteras 2024; Sethi et al. 2024). The activation of Nrf2 protects against mt dysfunction in neurodegenerative diseases, cardiovascular disorders, and metabolic syndromes by preventing ROS‐induced mt damage. Building on its role as a key energy sensor, AMPK not only activates transcription factors like PGC‐1α, and FOXO3 but also establishes a broader stress‐adaptive transcriptional program (Rakshe et al. 2024). PGC‐1α enhances the transcription of nuclear‐encoded mt genes involved in OXPHOS, mtDNA replication, and dynamics. Concurrently, AMPK‐mediated mediated upregulation of Nrf2 links mt biogenesis to antioxidant defense ensuring that increased mt activity occurs under conditions of controlled ROS levels (Song et al. 2024).

FOXO3a also plays a crucial role in linking mt biogenesis to antioxidant defense by activating genes involved in oxidative stress response, including SOD2 and catalase, while simultaneously regulating mitophagy through BNIP3 and LC3B expression. AMPK and Sirtuins (SIRT1, SIRT3) activate FOXO3a, reinforcing its role in mt quality control and cellular adaptation to stress (Zhu et al. 2024). Additionally, PPARs, particularly PPARγ and PPARδ, contribute to mt biogenesis and redox homeostasis by regulating fatty acid oxidation, mt DNA maintenance, and ROS detoxification. PPARs interact with Nrf2, further strengthening the antioxidant and metabolic response to mt stress. Sirtuins, a family of NAD+‐dependent deacetylases, play a key role in regulating both mt biogenesis and nuclear antioxidant signaling. SIRT1 enhances PGC‐1α activity, leading to increased mt gene expression and antioxidant defense, while SIRT3, which is localized within the mt, deacetylates and activates SOD2, improving ROS detoxification (Fiorenza et al. 2024; Palomba et al. 2024). The interplay between Nrf2 and NF‐κB is particularly significant in this context, as NF‐κB regulates inflammatory responses that can either promote or suppress mt resilience. Under chronic inflammation, excessive NF‐κB activation suppresses Nrf2 signaling, increasing oxidative stress and mt dysfunction. However, in acute stress conditions, Nrf2 activation dampens NF‐κB‐driven inflammation, fostering cytoprotection and metabolic adaptation (Das et al. 2025; Pant et al. 2024). Nuclear antioxidant responses to mt stress rely on an intricate network of transcription factors, metabolic sensors, and redox regulators (Gureev et al. 2024). Furthermore, the crosstalk between nuclear antioxidants and mt biogenesis, involving AMPK, PGC‐1α, FOXO3a, PPARs, Nrf2, and sirtuins, ensures that mt function is preserved under conditions of metabolic stress.

At physiological levels, ROS function as secondary messengers, modulating pathways such as AMPK‐PGC1α signaling to promote mt biogenesis (Sachdev et al. 2023). Transient fluctuations in ROS contribute to redox‐sensitive signaling, facilitating Nrf2‐driven antioxidant defenses and stabilizing HIF1α under hypoxic conditions. However, excessive or sustained ROS generation surpasses the cellular antioxidant capacity, resulting in oxidative stress, lipid peroxidation, protein modifications, and mtDNA damage, ultimately triggering apoptotic or pathological processes (Barzegari et al. 2022). The mt antioxidant defense system, encompassing enzymes such as superoxide dismutase, is essential for maintaining redox equilibrium and preventing oxidative dysregulation (Chidambaram et al. 2024). This bidirectional redox communication between mt and the nucleus underscores the delicate balance between ROS as regulators of cellular homeostasis and as potential contributors to oxidative pathology, highlighting the necessity of evaluating their levels, persistence, and detoxification capacity in the context of cellular function and disease progression. The transcription factor Nrf2, activated by oxidative stress, translocates to the nucleus, inducing genes involved in antioxidative and anti‐inflammatory pathways. Nrf2 links mt biogenesis with antioxidant functions (Baldelli et al. 2014; Piantadosi and Suliman 2012). In addition to its role in oxidative stress responses, Nrf2 is believed to coordinate mitophagy and the removal of damaged mtDNA, preventing the accumulation of defective mt genomes. This process ensures that only healthy mt persist, maintaining cellular efficiency and reducing oxidative burden. Nrf2 is thought to regulate the removal of misfolded proteins and damaged mt, although the networks connecting quality control of proteins, mitophagy, and mt biogenesis are less characterized (Jain et al. 2010; Kwak et al. 2003). The positive feedback loop involving AMPK, PGC‐1α, FOXO3a, PPARs, Nrf2, and sirtuins coordinates the expression of survival genes and promotes anti‐inflammatory actions by inhibiting NF‐κB, a key regulator of inflammatory responses (Salminen et al. 2011).

The PPARγ coactivator 1α protein (PGC‐1α) is a crucial regulator of the mt biogenesis gene network (Karakaidos and Rampias 2020; Scarpulla et al. 2012). The AMP‐activated protein kinase (AMPK), which belongs to the serine/threonine category, acts to restrain pathways that consume ATP while promoting those that generate ATP. Its action includes stimulating processes such as mt biogenesis, fatty acid oxidation, and glycolysis (Shirwany and Zou 2014). Upon oxidation, AMPK undergoes conformational changes, leading to its autophosphorylation and activation in an AMP‐dependent or AMP‐independent manner (Hagenbuchner and Ausserlechner 2013; Wu and Wei 2012). Phosphorylated AMPK activates transcription factors PGC‐1α and FOXO3a, which translocate to the nucleus, binding both antioxidant and autophagy promoters (Chiribau et al. 2008; Kops et al. 2002; Wu et al. 2014). PGC‐1α orchestrates mt biogenesis by co‐activating multiple transcription factors, including NRFs, estrogen‐related receptor alpha (ERRα), and PPARs. Through this coordinated activity, PGC‐1α, integrates metabolic and oxidative stress signals to fine‐tune mt function and antioxidant defense (Kemper et al. 2014; Polvani et al. 2012; Rius‐Pérez et al. 2020; Scarpulla et al. 2012). AMPK also inhibits NF‐κB by phosphorylating TSC1/2, which keeps mTOR inactive. Mt biogenesis is closely coordinated with antioxidants, anti‐inflammatory proteins, and autophagy to support energy‐demanding cell repair processes. This network recognizes ROS as byproducts of mt activity, requiring mechanisms for ROS management (Demirovic and Rattan 2013). Mild oxidative stress and energy‐demanding processes stimulate mt biogenesis, resulting in a larger, healthier mt population (López‐Lluch et al. 2006; Safdar et al. 2011). The AMPK, PGC‐1α, NRF2 axis serves as a key regulatory node that integrates mt quality control, redox homeostasis, and metabolic adaptation, ensuring cellular resilience under stress conditions.

Mt dynamics–shaped by redox cues–serve as a critical control point for cellular adaptation to metabolic and oxidative stress. For example. ROS modulate the mt network remodeling by influencing fission and fusion events, and by regulating mitophagy to ensure the selective removal of damaged mt and preserving overall mt function. This dynamic remodeling is tightly coordinated with the nuclear antioxidant response, mediated by key regulators such as Nrf2 and PGC‐1α, which together help sustain metabolic and energetic stability. Importantly, the balance between physiological ROS signaling and pathological oxidative stress is highly sensitive—its disruption can trigger mt dysfunction, widespread cellular damage, and the onset of diseases. A deeper understanding of this redox‐mt axis is essential for designing interventions that promote mt resilience and protect against oxidative stress‐related pathologies.

## 7 Summary and Future Directions

MtDNA is essential for cellular energy production, encoding core subunits of the OXPHOS machinery. Mutations in mtDNA can impair ATP production and elevate ROS levels, triggering a cycle of oxidative stress and cellular damage. To maintain mtDNA integrity, cells rely on multiple quality control mechanisms. Damaged or dysfunctional mt are selectively eliminated through mitophagy, a process tightly coordinated to mt fusion and fission to segregate and remove impaired mt components. In parallel, mt surveillance systems monitor both protein folding and structural integrity of the mt network, working to preserve mtDNA stability and ensure optimal mt function under both homeostatic and stress conditions.

Mt dynamics, specifically fission and fusion are critical for maintaining mt stability by regulating its distribution, copy number, and heteroplasmy across the mt network. These processes also interface with mitophagy, to control mtDNA turnover and removal of damaged genomes. Although fusion or fission are mechanistically distinct, disruption of either results in similar defects in mtDNA maintenance, suggesting potential overlap or compensation in their regulatory roles. Fission has been linked to the initiation of mtDNA replication, whereas fusion supports the uniform distribution of replication machinery components. However, the precise mechanisms by which these dynamics influence mtDNA integrity remain to be fully elucidated.

Thus, continued investigation into the mechanistic relationship between fusion, fission, and mitophagy in mtDNA maintenance is crucial. Insights from both basic research and clinical studies will enhance our understanding of mt dysfunction and its role in disease progression. A comprehensive understanding of these processes will facilitate the development of novel therapeutic strategies aimed at restoring mtDNA copy number and eliminating deleterious mtDNA mutations, ultimately mitigating mt dysfunction‐associated diseases. Clarifying how mitophagy selectively discriminates between defective and functional mtDNA is essential, as the persistence of deleterious mutations may undermine mt integrity and contribute to disease.

Recent findings suggest that oxidative damage may not be the primary driver of mtDNA mutations. TFAM helps shield mtDNA, and robust antioxidant systems efficiently detoxify ROS, limiting direct oxidative insults. Instead emerging evidence points to spontaneous nucleotide modifications, particularly deamination, as a dominant mutational mechanism. Despite this shift in understanding, several critical uncertainties remain. The nature, frequency, and distribution of these spontaneous modifications across the mt genome are not fully defined, nor is it clear whether certain regions are more vulnerable. The efficiency and specificity of mt BER‐the primary pathway‐also remain incompletely characterized. It is still unknown how effectively BER counters these mutations, or how unrepaired alterations might impact mtDNA replication and transcription. Crucially, the functional consequences of spontaneous mtDNA mutations remain underexplored, especially in the context of mt dysfunction and disease. A deeper understanding of these unresolved aspects is essential for clarifying the role of mt genome instability in aging, metabolic disorders and degenerative disease.

Despite earlier assumptions that mt DNA has limited repair capacity, we now recognize that mt possess a more extensive DNA repair system than previously assumed. BER serves as the principal pathway for maintaining mtDNA integrity, particularly in response to oxidative damage. However, emerging evidence suggests that other nuclear‐derived repair pathways–such as mismatch repair and homologous recombination‐may contribute to mtDNA maintenance, especially under conditions of stress or as a result of double strand break damage. However, the roles of mismatch repair and homologous recombination in the mt remain incompletelty understood. Proteins traditionaly associated with MMR, including MSH3, MSH6, and PMS2, have been detected within the mt, suggesting that some mismatch repair activity may occur within the organelle. Although, it remains unclear whether mismatch repair functions in mtDNA repair in the same manner as in the nucleus or if it specifically targets oxidative lesions rather than canonical replication mismatches. Additionally, efficiency and scope of MMR in correcting base mismatches and resolving insertion–deletion loops in mtDNA remain largely uncharacterized. Understanding the contributions of these lesser‐known repair pathways will be critical for elucidating the full extent of mitochondrial genome maintenance and its implications for cellular homeostasis and disease.

MtDNA repair relies heavily on nuclear‐encoded enzymes, highlighting the functional interdependence between mt and nuclear genomes in safeguarding cellular integrity. Key proteins like Pol‐γ and APE1 not only function in the mt but also contribute to nuclear repair, underscoring the evolutionary coordination of these systems. Mt import many of these enzymes from the nucleus to manage mtDNA damage, yet the mechanisms governing their translocation and regulation remain incompletely understood. It is not fully established whether these repair proteins are constitutively present in the mt or are recruited in response to specific stress signals such as oxidative stress, or mitochondrial UPRmt activation. Moreover, how these enzymes recognize mtDNA lesions, and whether their recruitment is modulated by cellular conditions like redox state or metabolic shifts, is unclear. A better understanding of these processes will provide critical insights into how cells prevent the accumulation of mtDNA mutations, ultimately mitigating mt dysfunction and disease progression.

The fidelity of mtDNA replication has emerged as a crucial determinant influencing its mutational burden, yet the cellular mechanisms that detect and respond to mtDNA damage remain poorly defined and questions remain regarding how mtDNA damage is recognized and its repair coordination. It is also not yet clear whether mtDNA damage sensors function autonomously within mt or rely on nuclear signaling cascade for activation. Additionally, the mechanisms by which damaged mtDNA is identified and selectively targeted for repair, degradation, or segregation remain poorly characterized.

While oxidative damage, repair mechanisms, and replication fidelity are central to mt genome stability, epigenetic regulation has emerged as a crucial yet understudied aspect of mt function. Unlike nuclear DNA, mtDNA lacks canonical histones, raising key questions about how epigenetic regulation influences mtDNA accessibility, transcription, and stability. Evidence suggests that DNA methylation and post‐translational modifications of TFAM influence mtDNA replication and transcription, yet the extent, specificity, and functional consequences of these modifications remain poorly understood. Another layer of mt epigenetic regulation involves TFAM and other histone‐like proteins that bind mtDNA and influence nucleoid structure and transcriptional activity. Post‐translational modifications of TFAM, such as acetylation and phosphorylation have been implicated in changes in mtDNA compaction and gene expression. However, it remains unclear how these modifications are modulated in response to cellular stress, or whether they act as signals for repair, degradation or long‐term transcriptional reprogramming. These knowledge gaps highlight the broader need to define the functional consequences of mt epigenetic changes– including TFAM modifications, mtDNA methylation, and non‐coding RNA activity, particularly given their emerging roles in neurodegeneration, cancer, and metabolic diseases. Clarifying these interdependent mechanisms will not only deepen our understanding of mt dysfunction but also reveal new opportunities for intervention in a wide range of mt‐related disorders.

**TABLE 1:** Interconnected roles of mt and the nucleus in cellular functions: Dynamic communication and responses to stress and metabolic demands.

Function	Actions	Role of Mt	Other cellular signals	Role of nucleus	Ref.
Stress response	ROS signaling	Mt generate ROS as superoxide anions, driven by hypoxia and defects in mt respiration, with enzymes like ETC complexes I and III contributing to ROS production.	Elevated ROS levels induce lipid peroxidation, generating electrophiles that oxidize cysteine residues and impair protein function.	The nucleus detects mt ROS signals, activating antioxidant response genes and regulating redox‐sensitive events.	Selivanov et al. (2011) and Scholtes and Giguère (2021)
Mt metabolism & biogenesis	DNA transcription	(1) Changes in AMP/ATP ratio, triggered by cellular stress, activate AMPK, signaling the nucleus to enhance mt biogenesis. (2) TFAM binds to mtDNA, promoting replication & transcription, a key step in mt biogenesis.	Nutritional status: amino acids & glucose influence mTOR activation.	AMPK activates PGC‐1α, which coactivates NRF1 and NRF2 for mt biogenesis. TFAM is activated by AMPK, in response to OXPHOS defects in the nucleus. Low nutrient levels and a high AMP/ATP ratio inhibit AMPK and mTOR. mTORC1 is activated by high nutrient levels, promotes protein and lipid synthesis through S6K1, 4E‐BP1, and SREBP activation.	Ling et al. (2020)
	Fission & Fusion of mt	Mt undergoes fission to isolate damaged parts for degradation and fusion to share contents, supporting repair and maintaining a healthy network.	Changes in cellular redox status and ATP levels regulate mitochondrial dynamics, with high ROS levels triggering fission and increased ATP promoting fusion to optimize energy efficiency and maintain cellular health.	The nucleus produces proteins essential for fission (Drp1 and Fis1) and fusion (Mfn1, Mfn2, and OPA1), adapting mt structure as needed.	Ihenacho et al. (2023)

## Data Availability

Data sharing not applicable to this article as no datasets were generated or analysed during the current study.
